# Serum uric acid is independently associated with impaired nitroglycerine-induced vasodilation of the brachial artery in women

**DOI:** 10.1038/s41440-024-01972-5

**Published:** 2024-11-14

**Authors:** Tatsuya Maruhashi, Masato Kajikawa, Shinji Kishimoto, Takayuki Yamaji, Takahiro Harada, Aya Mizobuchi, Shunsuke Tanigawa, Farina Mohamad Yusoff, Yukiko Nakano, Kazuaki Chayama, Ayumu Nakashima, Chikara Goto, Yukihito Higashi

**Affiliations:** 1 Department of Regenerative Medicine, Division of Radiation Medical Science, Research Institute for Radiation Biology and Medicine, Hiroshima University, 1-2-3 Kasumi, Minami-ku, Hiroshima, 734-8553 Japan; 2 Division of Regeneration and Medicine, Medical Center for Translational and Clinical Research, Hiroshima University Hospital, 1-2-3 Kasumi, Minami-ku, Hiroshima, 734-8551 Japan; 3 Department of Cardiovascular Medicine, Graduate School of Biomedical and Health Sciences, Hiroshima University, 1-2-3 Kasumi, Minami-ku, Hiroshima, 734-8551 Japan; 4 Department of Medicine and Molecular Science, Hiroshima University Graduate School of Biomedical Sciences, Hiroshima University, 1-2-3 Kasumi, Minami-ku, Hiroshima, 734-8551 Japan; 5 Department of Stem Cell Biology and Medicine, Graduate School of Biomedical and Sciences, Hiroshima University, 1-2-3 Kasumi, Minami-ku, Hiroshima, 734-8551 Japan; 6 Department of Rehabilitation, Faculty of general Rehabilitation, Hiroshima International University, 555-36, Kurosegakuendai, Higashihiroshima, 739-2695 Japan

**Keywords:** Vascular smooth muscle cell, Nitroglycerine-induced vasodilation, Uric acid, Atherosclerosis

## Abstract

Experimental and clinical studies have suggested atherosclerotic effects of uric acid (UA) on vascular smooth muscle cells (VSMCs). Nitroglycerine-induced vasodilation (NID), a control test for flow-mediated vasodilation, can be used as a possible marker of VSMC dysfunction. However, there is little information on the association between UA and NID. Therefore, we investigated the association between serum UA levels and NID according to sex. We measured NID of the brachial artery in 598 women (mean age: 66.2 ± 12.0 years) and 1008 men (mean age: 59.0 ± 18.0 years). In women, the mean serum UA level was 5.06 ± 1.24 mg/dL. Serum UA levels were negatively correlated with NID (*p* < 0.001), and NID significantly decreased with increasing serum UA levels (≤4.0 mg/dL, 13.4 ± 6.4%; 4.0 to ≤5.0 mg/dL, 11.4 ± 5.3%; 5.0 to ≤6.0 mg/dL, 10.8 ± 5.7%; >6.0 mg/dL, 9.7 ± 5.7%; *p* < 0.001). The prevalence of VSMC dysfunction, defined as NID < 8.4%, the division points for the lowest and middle tertiles of NID in women, increased with increasing serum UA levels ( ≤ 4.0 mg/dL, 23.3%; 4.0 to ≤5.0 mg/dL, 30.9%; 5.0 to ≤6.0 mg/dL, 36.4%; >6.0 mg/dL, 44.6%; *p* < 0.001). Multiple logistic regression analysis showed a significant association between serum UA levels and VSMC dysfunction (odds ratio, 1.21; 95% confidence interval, 1.02─1.43; *p* = 0.03). There was no interaction between age (<50 or ≥50 years) and the effect of serum UA levels on VSMC dysfunction (*p* interaction = 0.88). In contrast, no association was observed between serum UA levels and NID in men. Serum UA levels were significantly associated with VSMC dysfunction as assessed by NID in women.

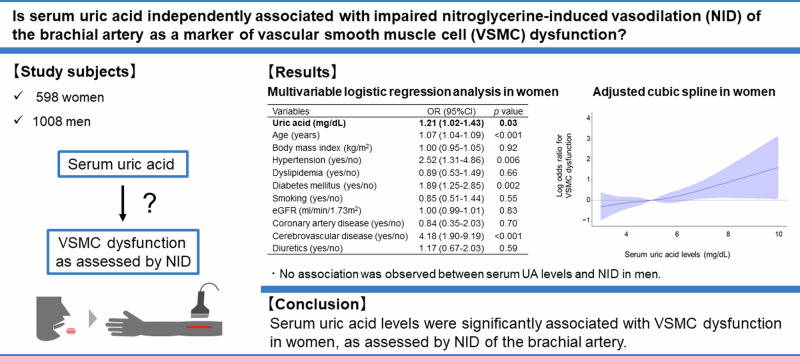

## Introduction

Uric acid (UA) is the end product of purine metabolism catalyzed by xanthine oxidase in humans. Although it remains a matter of debate whether UA is an independent causal risk factor for atherosclerosis, epidemiological studies have shown significant associations of serum UA levels with atherosclerosis and cardiovascular disease [1–4]. The medial layers of the arteries, composed mainly of vascular smooth muscle cells (VSMCs) and connective tissue elements, play an important role in the progression of arterial stiffness [5]. The functional and structural changes in the medial layers, including high tone of VSMCs, thickening of the intima-media layer, and abnormalities of connective tissue elements, contribute to arterial stiffening. In vitro studies have shown that UA taken up by human VSMCs via urate transporters causes VSMC proliferation and inflammation in a UA concentration-dependent manner [6, 7]. In addition, recent clinical studies have suggested that UA is causally related to the progression of arterial stiffness [8–10]. These findings suggest that UA taken up by VSMCs contributes to the progression of arterial stiffness through functional and structural changes in VSMCs, leading to the progression of arterial stiffness.

Nitroglycerine-induced vasodilation (NID) of the brachial artery, an index of endothelium-independent vasodilation, is assessed by measuring changes in brachial artery diameter after nitroglycerine administration. Measurements of NID have been used as a control test for flow-mediated vasodilation (FMD), an index of endothelial function, to ensure that the vascular response to reactive hyperemia is not affected by the reactivity of VSMCs to nitric oxide (NO) but is truly a consequence of endothelial function. However, recent studies have shown that NID of the brachial artery per se is impaired in patients with cardiovascular risk factors or a history of cardiovascular disease, that brachial artery NID is significantly correlated with coronary artery NID, a possible prognostic marker of atherosclerosis [11, 12], and that patients with impaired NID of the brachial artery have a higher risk of cardiovascular events independent of FMD [13, 14]. These findings suggest that NID of the brachial artery can be used as a vascular marker of atherosclerosis. Although the exact mechanisms of NID impairment remain to be elucidated, functional and structural abnormalities in VSMCs may be involved in NID impairment [13]. Considering the significant association between UA and the progression of arterial stiffness, UA may also be associated with impairment of NID of the brachial artery by altering the function and structure of VSMCs. However, there is little information on the association between UA and NID of the brachial artery.

Previous studies have shown that the effects of UA on atherosclerosis and cardiometabolic disease are stronger in women than in men [15]. Several cross-sectional studies have shown a stronger association between UA and arterial stiffness as assessed by brachial-ankle pulse wave velocity (baPWV) or cardio-ankle vascular index (CAVI) in women than in men [16–18]. Regarding endothelial function, Tanaka et al. showed stronger cross-sectional and longitudinal associations between UA and FMD in women than in men [19]. These findings suggest a sex difference in the association between UA and vascular function. However, a sex difference in the association between UA and NID of the brachial artery is unclear. Therefore, in this study, we investigated the association between serum UA levels and NID of the brachial artery according to sex in a large number of subjects.

Point of view

- Clinical relevance
  Serum UA levels were significantly associated with VSMC dysfunction, as assessed by NID of the brachial artery in women. UA may contribute to both functional and structural changes in VSMCs in women.
- Future direction
  Further studies are needed to elucidate the underlying mechanisms behind the sex difference in the association between UA and NID of the brachial artery. In addition, it is essential to determine whether urate-lowering therapies, such as urate transporter inhibitors or xanthine oxidoreductase inhibitors, can improve NID in women.
- Consideration for the Asian population
  Hyperuricemia should be recognized as a risk factor for atherosclerosis in Asian women. Aggressive management strategies for hyperuricemia are crucial for future public health initiatives in Asia, where the prevalence of hyperuricemia is increasing.

## Methods

### Subjects

This study was a cross-sectional study. Between July 2007 and December 2019, 2659 subjects were recruited for NID measurement for cardiovascular risk assessment from patients who visited the cardiovascular outpatient clinic and subjects who underwent health screening examinations with consent for vascular function assessment at Hiroshima University Hospital. Data were retrospectively analyzed. We excluded participants receiving nitrate treatment (*n* = 129) and those with malignant disease (*n* = 43), collagen disease (*n* = 53), atrial fibrillation (*n* = 170), secondary hypertension (*n* = 357), and missing information on serum UA levels (*n* = 301). These patients were excluded because NID may be affected by anticancer drugs, inflammation, steroids, anti-inflammatory drugs, irregular shear stress, and hormones such as aldosterone. Finally, 598 women (mean age: 66.2 ± 12.0 years) and 1008 men (mean age: 59.0 ± 18.0 years) were enrolled in this study. Hypertension was defined as treatment with oral antihypertensive drugs or systolic blood pressure of more than 140 mm Hg and/or diastolic blood pressure of more than 90 mm Hg in a sitting position on at least 3 different occasions without medication [20]. Diabetes was defined according to the American Diabetes Association recommendation [21]. Dyslipidemia was defined according to the third report of the National Cholesterol Education Program [22]. We defined smokers as those who had ever smoked. Cardiovascular disease included coronary artery disease (CAD) and cerebrovascular disease. CAD included angina pectoris, myocardial infarction, and unstable angina. Cerebrovascular disease included ischemic stroke, hemorrhagic stroke, and transient ischemic attack. The estimated glomerular filtration rate (eGFR) was calculated using the Japanese eGFR equation [23]. Chronic kidney disease (CKD) was defined as eGFR <60 mL/min/1.73 m^2^ [24]. Measurement of NID was performed without withholding medications. Serum UA levels were measured using the uricase-peroxidase method (JCA-BM6010; JEOL Ltd., Tokyo, Japan). Serum UA, total cholesterol (TCHO), triglycerides (TG), high-density lipoprotein cholesterol (HDL-C), low-density lipoprotein cholesterol (LDL-C), and glucose levels were measured in mg/dL. The following factors were used to convert to the International System of Units: 1 mg/dL = 0.02586 mmol/L for TCHO, HDL-C, and LDL-C, 0.01129 mmol/L for TG, 0.05551 mmol/L for glucose, and 59.48 μmol/L for UA. This study was performed in accordance with the 1975 Declaration of Helsinki. The ethical committee of our institution (Hiroshima University Hospital Institutional Review Board) approved the study protocol. Written informed consent for participation in the study was obtained from all participants. The protocol was registered in the University Hospital Medical Information Network Clinical Trials Registry (UMIN000039512).

### Study protocol

Subjects fasted the previous night for at least 8 h and abstained from consuming caffeine, alcohol, and antioxidant vitamins and from smoking on the day of the examination. The subjects were kept in the supine position in a quiet, dark, air-conditioned room (constant temperature, 23–26 °C) throughout the study. A 23-gauge polyethylene catheter was inserted into the left deep antecubital vein to obtain blood samples. At least 20 min after maintaining the supine position, NID measurements were performed by skilled and trained physicians or sonographers without knowledge of the baseline clinical characteristics of the subjects.

### NID measurement

A high-resolution linear artery transducer was coupled to computer-assisted analysis software (UNEXEF18G, UNEX Co., Nagoya, Japan) that used an automated edge-detection system to measure the brachial artery diameter. The response of brachial artery diameter to nitroglycerine was used to assess endothelium-independent vasodilation. After acquiring baseline rest images of the brachial artery for 30 s, a sublingual tablet (75 μg nitroglycerine) was administered, and images of the artery were continuously recorded until the dilation reached a plateau after nitroglycerine administration. A few minutes after the administration of the nitroglycerine tablet, we carefully checked in the mouth to confirm that the nitroglycerine tablet had been dissolved and absorbed. Subjects in whom the nitroglycerine tablet did not dissolve during the measurement were not included in this study. NID was calculated as the percentage change in peak vessel diameter from the baseline value. The percentage of NID [(peak diameter—baseline diameter)/baseline diameter] was used for analysis.

### Statistical analysis

All reported *p* values were 2-sided, and a *p* value of <0.05 was considered statistically significant. Continuous variables were summarized as mean ± standard deviation (SD) and were compared using the unpaired Student’s *t* test or analysis of variance for multiple groups. Categorical variables were presented as frequencies and percentages and were compared using the chi-squared test. The Pearson product-moment correlation coefficient was calculated to examine the relationship between serum UA levels and NID. The division points for the lowest and middle tertiles of NID were 8.4% in women and 9.9% in men. Therefore, VSMC dysfunction was defined as NID < 8.4% for women and NID < 9.9% for men. The Cochran-Amitage trend test was used to assess the trend of ordered categorical variables for the association between serum UA levels and the proportion of subjects with VSMC dysfunction. Multiple logistic regression analysis was performed to identify independent variables associated with VSMC dysfunction. UA level, age, body mass index (BMI) or BMI ≥ 25, hypertension, dyslipidemia, diabetes mellitus, smoking, eGFR or CKD, CAD, cerebrovascular disease, and diuretic use were entered into the model. The effect of serum UA levels on VSMC dysfunction was assessed as a continuous variable using cubic spline curves with 5.0 mg/dL as the references. Age, BMI, hypertension, dyslipidemia, diabetes mellitus, smoking, eGFR, CAD, cerebrovascular disease, and diuretic use were entered into the model. The effects of serum UA levels on VSMC dysfunction were estimated in women aged <50 years and ≥50 years as a subgroup analysis. Data were processed using JMP version 17 (SAS Institute, Cary, NC) and R software version 3.5.1 and version 3.6.2 (R Foundation for Statistical Computing, Vienna, Austria).

## Results

### Baseline clinical characteristics of women

The baseline clinical characteristics of the women are summarized in Table 1. Of the 598 female subjects, 498 (83.4%) had hypertension, 475 (79.6%) had dyslipidemia, 191 (32.1%) had diabetes mellitus, 176 (29.5%) had CKD, 30 (5.1%) had CAD, 36 (6.1%) had cerebrovascular disease, and 116 (19.5%) were smokers. The mean serum UA level was 5.06 ± 1.24 mg/dL (median, 4.9 mg/dL; interquartile range, 4.2–5.8 mg/dL; range, 2.0–10.1 mg/dL). The mean NID was 11.2 ± 5.8% (range, 0.0–33.8%).

**Table 1:** Clinical characteristics of the subjects

Variables	Women	Men
	(*n* = 598)	(*n* = 1008)
Age, y	66.2 ± 12.0	59.0 ± 18.0
Body mass index, kg/m^2^	23.3 ± 4.1	24.0 ± 3.6
Systolic blood pressure, mm Hg	131.8 ± 18.8	129.3 ± 18.1
Diastolic blood pressure, mm Hg	76.0 ± 12.0	77.3 ± 12.3
Heart rate, bpm	70.4 ± 11.3	69.1 ± 29.1
Total cholesterol, mg/dL	201.0 ± 36.9	186.5 ± 36.9
Triglycerides, mg/dL	125.1 ± 73.6	149.7 ± 129.4
HDL-cholesterol, mg/dL	63.8 ± 16.9	57.2 ± 16.0
LDL-cholesterol, mg/dL	116.1 ± 33.4	105.8 ± 32.5
Glucose, mg/dL	112.8 ± 37.5	113.8 ± 39.8
Uric acid, mg/dL	5.06 ± 1.24	6.09 ± 1.34
eGFR, mL/min/1.73 m^2^	70.3 ± 27.6	72.2 ± 20.8
Smoking, n (%)	116 (19.5)	743 (74.0)
Comorbidities		
Hypertension, n (%)	498 (83.4)	768 (76.3)
Dyslipidemia, n (%)	475 (79.6)	685 (68.0)
Diabetes mellitus, n (%)	191 (32.1)	277 (27.5)
Coronary artery disease, n (%)	30 (5.1)	202 (20.2)
Cerebrovascular disease, n (%)	36 (6.1)	86 (8.6)
Chronic kidney disease, n (%)	176 (29.5)	238 (23.6)
Medication use		
Antihypertensive drug, n (%)	432 (72.9)	656 (65.9)
Diuretics, n (%)	75 (12.6)	139 (14.0)
Lipid-lowering drugs, n (%)	287 (48.3)	364 (36.6)
Hypoglycemic drugs	132 (22.2)	187 (18.8)

*HDL* indicates high-density lipoprotein, *LDL* low-density lipoprotein, *eGFR* estimated glomerular filtration rate

### Relationships between serum UA levels and NID in women

**Fig. 1:**
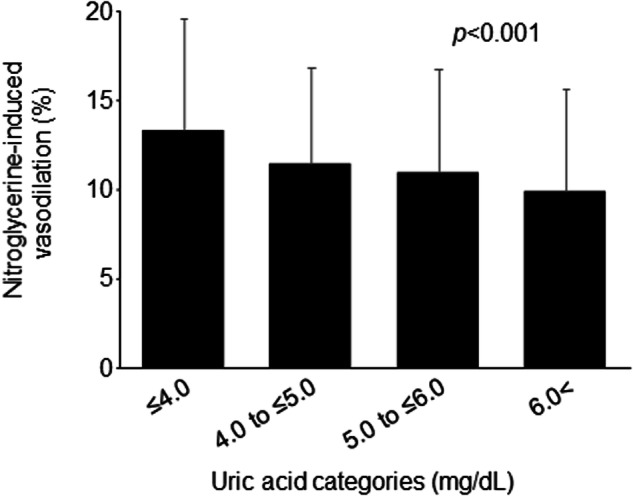
Bar graphs show nitroglycerine-induced vasodilation of the brachial artery categorized according to serum uric acid levels in women

**Fig. 2:**
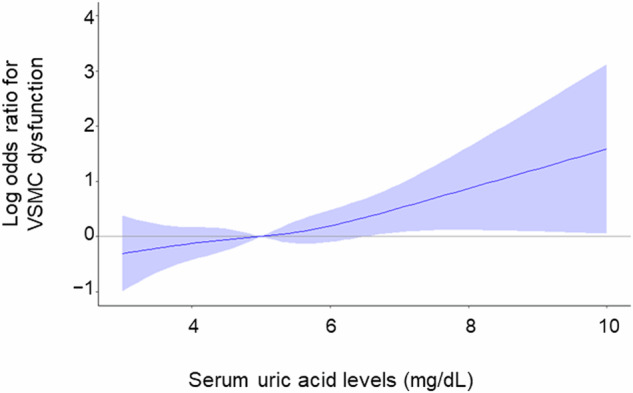
Adjusted cubic spline of the relationship between serum uric acid levels and vascular smooth muscle cell (VSMC) dysfunction in women. The adjusted model includes age, body mass index, hypertension, dyslipidemia, diabetes mellitus, smoking, estimated glomerular filtration rate, coronary artery disease, cerebrovascular disease, and diuretic use

Univariate regression analysis showed that serum UA levels were negatively correlated with NID (r = –0.20, *p* < 0.001). Subjects were categorized according to serum UA levels (Table 2). NID significantly decreased with increasing serum UA levels (≤4.0 mg/dL, 13.4 ± 6.4%; 4.0 to ≤5.0 mg/dL, 11.4 ± 5.3%; 5.0 to ≤6.0 mg/dL, 10.8 ± 5.7%; >6.0 mg/dL, 9.7 ± 5.7%; *p* < 0.001, Fig. 1). The prevalence of VSMC dysfunction increased with increasing serum UA levels (≤4.0 mg/dL, 23.3%; 4.0 to ≤5.0 mg/dL, 30.9%; 5.0 to ≤6.0 mg/dL, 36.4%; >6.0 mg/dL, 44.6%; *p* < 0.001). Multiple logistic regression analysis revealed that serum UA levels were significantly associated with a higher risk of VSMC dysfunction after adjusting for other confounders (odds ratio [OR], 1.21; 95% confidence interval [CI], 1.02 to 1.43; *p* = 0.03) (Table 3). We evaluated the effect of serum UA levels on VSMC dysfunction by using cubic spline curves. The cubic spline curve showed that the ORs for VSMC dysfunction increased linearly with increasing serum UA levels (Fig. 2). The cutoff value of serum UA levels associated with VSMC dysfunction was determined according to the highest Youden index from a receiver operating characteristic (ROC) curve to diagnose VSMC dysfunction. The optimal cutoff value of serum UA levels for VSMC dysfunction was 4.8 mg/dL. We divided the participants into two groups: subjects <50 years of age (*n* = 61) and subjects ≥50 years of age (*n* = 537). There was no interaction between age (<50 or ≥50 years) and the effect of serum UA levels on VSMC dysfunction (*p* interaction = 0.88) (Supplementary Fig. 1).

**Table 2:** Clinical characteristics according to serum uric acid levels in women

Variables	Uric acid categories, mg/dL				*p* value
	≤4.0	4.0 to ≤5.0	5.0 to ≤6.0	6.0<	
	(*n* = 91)	(*n* = 221)	(*n* = 166)	(*n* = 120)	
Uric acid, mg/dL	3.32 ± 0.51	4.48 ± 0.27	5.46 ± 0.29	6.87 ± 0.87	NA
Age, y	62.4 ± 13.2	66.0 ± 11.5	67.2 ± 10.6	68.0 ± 13.3	0.004
Body mass index, kg/m^2^	21.3 ± 3.0	23.0 ± 3.9	24.3 ± 4.2	24.1 ± 4.3	<0.001
Systolic blood pressure, mm Hg	129.8 ± 18.3	131.4 ± 18.5	132.9 ± 19.6	132.5 ± 18.8	0.59
Diastolic blood pressure, mm Hg	75.6 ± 11.7	75.8 ± 12.0	77.6 ± 12.3	74.4 ± 11.4	0.15
Heart rate, bpm	69.8 ± 9.1	71.4 ± 11.8	69.1 ± 10.6	70.7 ± 12.5	0.20
Total cholesterol, mg/dL	204.6 ± 42.3	199.5 ± 37.8	203.5 ± 35.4	197.6 ± 32.6	0.40
Triglycerides, mg/dL	111.6 ± 63.9	117.7 ± 71.3	134.5 ± 76.1	136.6 ± 78.9	0.01
HDL-cholesterol, mg/dL	68.3 ± 19.5	65.2 ± 16.7	61.1 ± 15.5	61.4 ± 16.2	0.002
LDL-cholesterol, mg/dL	120.3 ± 37.6	113.8 ± 34.0	120.2 ± 31.0	111.4 ± 31.2	0.07
Glucose, mmol/L	109.8 ± 28.1	112.2 ± 35.1	112.1 ± 41.4	117.2 ± 41.8	0.53
eGFR, mL/min/1.73 m^2^	79.9 ± 14.0	72.0 ± 17.9	67.6 ± 16.4	63.6 ± 51.0	<0.001
Hypertension, n (%)	58 (64.4)	182 (82.4)	153 (92.2)	105 (87.5)	<0.001
Dyslipidemia, n (%)	62 (68.9)	171 (77.4)	142 (85.5)	100 (83.3)	0.001
Diabetes mellitus, n (%)	23 (25.6)	79 (35.9)	49 (29.5)	41 (34.5)	0.25
Coronary artery disease, n (%)	3 (3.3)	13 (5.9)	5 (3.0)	9 (7.6)	0.26
Cerebrovascular disease, n (%)	5 (5.6)	14 (6.4)	10 (6.0)	7 (5.9)	0.99
Smoking, n (%)	16 (18.0)	39 (17.7)	34 (20.6)	27 (22.5)	0.71
Chronic kidney disease, n (%)	4 (4.4)	46 (20.9)	58 (34.9)	68 (57.1)	<0.001
Diuretics, n (%)	1 (1.1)	20 (9.1)	33 (20.0)	21 (17.7)	<0.001

*HDL* indicates high-density lipoprotein, *LDL* low-density lipoprotein, *eGFR* estimated glomerular filtration rate, *NA* not applicable

*p* values for comparisons across the uric acid categories were performed with ANOVA for continuous variables and χ^2^ test for categorical variables

**Table 3:** Multivariate analysis of the relation between vascular smooth muscle dysfunction and variables in women

Variables	Model 1		Model 2		Model 3		Model 4		Model 5	
	OR (95%CI)	*p* value	OR (95%CI)	*p* value	OR (95%CI)	*p* value	OR (95%CI)	*p* value	OR (95%CI)	*p* value
Uric acid (mg/dL)	1.31 (1.14–1.51)	<0.001	1.20 (1.04–1.40)	0.01	1.17 (0.98–1.39)	0.08	1.28 (1.02–1.43)	0.03	1.21 (1.02–1.43)	0.03
Age (years)	—	—	1.07 (1.05–1.09)	<0.001	1.06 (1.04–1.09)	<0.001	1.07 (1.04–1.09)	<0.001	1.07 (1.04–1.09)	<0.001
Body mass index (kg/m^2^)	—	—	—	—	1.00 (0.95–1.05)	0.92	—	—	1.00 (0.95–1.05)	0.92
Body mass index ≥25 (kg/m^2^)	—	—	—	—	—	—	0.98 (0.63–1.51)	0.98	—	—
Hypertension (yes/no)	—	—	—	—	2.49 (1.30–4.79)	0.006	2.55 (1.33–4.90)	0.005	2.52 (1.31–4.86)	0.006
Dyslipidemia (yes/no)	—	—	—	—	0.90 (0.54–1.50)	0.68	0.90 (0.54–1.50)	0.68	0.89 (0.53–1.49)	0.66
Diabetes mellitus (yes/no)	—	—	—	—	1.90 (1.26–2.87)	0.002	1.90 (1.26–2.86)	0.002	1.89 (1.25–2.85)	0.002
Smoking (yes/no)	—	—	—	—	0.85 (0.51–1.44)	0.56	0.85 (0.51–1.44)	0.55	0.85 (0.51–1.44)	0.55
eGFR (ml/min/1.73m^2^)	—	—	—	—	—	—	1.00 (0.99–1.01)	0.83	1.00 (0.99–1.01)	0.83
Chronic kidney disease (yes/no)	—	—	—	—	1.19 (0.76–1.86)	0.46	—	—	—	—
Coronary artery disease (yes/no)	—	—	—	—	0.81 (0.34–1.95)	0.64	0.84 (0.35–2.04)	0.70	0.84 (0.35–2.03)	0.70
Cerebrovascular disease (yes/no)	—	—	—	—	4.21 (1.92–9.24)	<0.001	4.16 (1.89–9.17)	<0.001	4.18 (1.90–9.19)	<0.001
Diuretics (yes/no)	—	—	—	—	1.16 (0.67–2.01)	0.61	1.17 (0.67–2.04)	0.58	1.17 (0.67–2.03)	0.59

*OR* odds ratio, *CI* confidence interval, *eGFR* estimated glomerular filtration rate

### Baseline clinical characteristics of men

The baseline clinical characteristics of the men are summarized in Table 1. Of the 1008 male subjects, 768 (76.3%) had hypertension, 685 (68.0%) had dyslipidemia, 277 (27.5%) had diabetes mellitus, 238 (23.6%) had CKD, 202 (20.2%) had CAD, 86 (8.6%) had cerebrovascular disease, and 743 (74.0%) were smokers. The mean serum UA level was 6.09 ± 1.34 mg/dL (median, 6.0 mg/dL; interquartile range, 5.3–6.9 mg/dL; range, 1.8 to 11.8 mg/dL). The mean NID was 12.4 ± 5.8% (range, 0.0–32.2%).

### Relationships between serum UA levels and NID in men

Univariate regression analysis showed no correlation between serum UA levels and NID (r = –0.004, *p* = 0.90). Subjects were categorized according to serum UA levels (Supplementary Table 1). There was no significant difference in NID among the four groups (≤5.0 mg/dL, 12.4 ± 6.0%; 5.0 to ≤6.0 mg/dL, 12.2 ± 5.4%; 6.0 to ≤7.0 mg/dL, 12.8 ± 5.9%; >7.0 mg/dL, 12.3 ± 6.2%; *p* = 0.63, Supplementary Fig. 2). There was no significant difference in the prevalence of VSMC dysfunction among the four groups (≤5.0 mg/dL, 31.3%; 5.0 to ≤6.0 mg/dL, 33.7%; 6.0 to ≤7.0 mg/dL, 32.0%; >7.0 mg/dL, 35.5%; *p* = 0.49). Multiple logistic regression analysis revealed no significant association between serum UA levels and VSMC dysfunction (OR, 1.04; 95% CI, 0.93 to 1.17; *p* = 0.47) (Supplementary Table 2). The cubic spline curve showed that there was no increase in ORs for VSMC dysfunction with increasing serum UA levels (Supplementary Fig. 3).

## Discussion

In the present study, we demonstrated that serum UA levels were negatively correlated with NID of the brachial artery, an index of VSMC function, and that serum UA levels were significantly associated with VSMC dysfunction, defined as NID < 8.4%, even after adjusting for other confounders in women. There was no interaction between age (<50 or ≥50 years) and the effect of serum UA levels on VSMC dysfunction. In contrast, no significant association was observed between serum UA levels and VSMC dysfunction in men. To our knowledge, this is the first study to demonstrate a significant association between serum UA levels and VSMC dysfunction as assessed by NID in women.

Recent clinical studies have shown that UA may contribute to the progression of arterial stiffness. Tomiyama et al. showed that UA was significantly associated with the progression of baPWV by analysis of data from middle-aged Japanese occupational cohort with a 16-year follow-up period [8]. Shina et al. reported that the progression of arterial stiffness as assessed by baPWV and CAVI was inhibited by 24-month treatment with febuxostat in patients with asymptomatic hyperuricemia [9]. In addition, Tanaka et al. reported that CAVI was improved by 24 weeks of treatment with dotinurad in hypertensive patients with asymptomatic hyperuricemia [10]. These findings suggest that UA is causally related to the progression of arterial stiffness. The medial layers, which are composed mainly of VSMCs and connective tissue elements, play a critical role in arterial stiffening. Therefore, UA may contribute to NID impairment through functional and structural changes in VSMCs.

Several cross-sectional studies in which sex differences in the association between serum UA levels and the presence of atherosclerosis were investigated have shown that the association between UA and atherosclerosis, including endothelial function as assessed by FMD, arterial stiffness as assessed by baPWV or CAVI, and carotid plaque, is stronger in women than those in men [16–18, 25]. Although, to our knowledge, there have been few studies in which sex differences in the association between serum UA levels and progression of atherosclerosis were longitudinally investigated, Tanaka et al. showed a stronger longitudinal association between UA and FMD in women than in men [19]. These findings suggest a sex difference in the association between UA and atherosclerosis. The association between serum UA levels and NID of the brachial artery has been investigated in several studies, and most previous studies have shown no significant association between serum UA levels and NID [26–30]. However, sex differences were not considered in most previous studies, despite the known difference in serum UA levels and differences in the effects of UA on cardiometabolic disease and atherosclerosis between men and women [15], making it difficult to investigate the exact association between serum UA levels and NID. A previous study showed a significant association between serum UA levels and NID in patients with diabetes and/or CAD [31]; however, both men and women were analyzed together, and participants receiving nitrate treatment were not excluded from that study. In the present study, we carefully excluded subjects receiving nitrate treatment and investigated the association between serum UA levels and NID separately in men and women. The results of the present study showed that serum UA levels, as well as age, hypertension, diabetes, and a history of cerebrovascular disease, were significantly associated with VSMC dysfunction in women but not in men. The cutoff value of serum UA levels associated with VSMC dysfunction derived from the ROC curve was 4.8 mg/dL in women. However, VSMC dysfunction, defined as NID < 8.4%, was determined by using the division point for the lowest and middle tertiles in this study population of women. Therefore, this cutoff value may not apply to other populations and should be interpreted with caution. The insignificant association between serum UA levels and NID in men observed in this study is consistent with the results of a previous study by Kato et al. showing that there was no significant difference in NID between male subjects with hyperuricemia and those without hyperuricemia, suggesting no significant association between UA and NID in men [32]. Epidemiological studies have shown that the effects of UA on CAD may be stronger in women than in men [33]. The results of this study support the previous findings and indicate the possibility that sex differences in the effects of UA on VSMC dysfunction may contribute to sex differences in the effects of UA on CAD. Further studies are needed to determine whether sex differences in the association between serum UA levels and NID contribute to a stronger association between UA and CAD in women than in men.

Although the exact mechanisms underlying NID impairment in patients with cardiovascular risk factors are not fully understood, it has been postulated that increased reactive oxygen species and inflammatory cytokines in VSMCs under pathological conditions may inhibit the activities of soluble guanylyl cyclase (sGC) and cGMP-dependent protein kinase in VSMCs, leading to an impaired vasodilatory response to administered nitroglycerine [13, 34]. In addition, a relative decrease in nitroglycerine-derived NO due to VSMC proliferation and limited relaxation due to an increased connective tissue matrix in thickened intima-media layers may contribute, in part, to NID impairment. The uptake of UA by VSMCs and the resulting functional and structural changes in VSMCs, including the attenuation of NO-sGC-cGMP signaling due to inflammation and VSMC proliferation, may contribute, in part, to the significant association between UA and NID impairment.

The reasons for the sex difference in the association between UA and VSMC dysfunction are unclear. One possible hypothesis is that high serum UA levels indicate high urate production with increased xanthine oxidase activity and generation of reactive oxygen species as by-products in women, which may lead to an impaired response of VSMCs to administered nitroglycerine [35]. Another possible hypothesis is that the expression and function of urate transporters in VSMCs differ between men and women, which may lead to sex differences in the association between UA and VSMC dysfunction. However, to our knowledge, there have been no studies in which the sex differences in the expression and function of urate transporters in VSMCs were investigated. Further studies are needed to investigate the mechanisms underlying sex differences in the association between UA and VSMC function.

This study has several limitations. First, a definitive causal relationship between UA and NID could not be established because of the cross-sectional design of this study. Further studies are needed to determine whether urate-lowering therapy with urate transporter inhibitors or xanthine oxidoreductase inhibitors can improve NID in women. Second, there were several differences in the cardiovascular risk conditions between men and women in this study. Serum UA levels are confounded by many cardiovascular risk and lifestyle factors. Although the multivariable analyses were corrected for several confounding factors when we investigated the association between serum UA levels and VSMC dysfunction, we cannot deny the possibility that the sex difference in the association between serum UA levels and VSMC dysfunction is influenced by the differences in cardiovascular risk conditions between the sexes. Third, the possibility of residual unmeasured confounding factors cannot be excluded. Information on lifestyle, diet, exercise habits, and urate-lowering agents was not available, and these factors were not considered in this study. Smoking was negatively associated with VSMC dysfunction in men in this study. Although we do not know the exact reason why smoking was negatively associated with VSMC dysfunction in men, 462 of the 743 smokers were ex-smokers, and ex-smokers may be health conscious. When unmeasured factors, including lifestyle, diet, and exercise habits, were considered in the multivariable analyses, the results regarding the association between smoking and VSMC dysfunction may change. In addition, a comparison of NID between patients on xanthine oxidoreductase inhibitors and those on urate transporter inhibitors may provide more specific conclusions regarding the effects of UA on VSMC function and the mechanisms underlying the association between UA and VSMC dysfunction.

### Perspective of Asia

The prevalence of gout and hyperuricemia has been increasing in Asia. The findings of the present study suggest that hyperuricemia should be regarded as a risk factor for atherosclerosis in Asian women and that aggressive management strategies for hyperuricemia are crucial for future public health initiatives. The effective management and treatment of hyperuricemia may improve VSMC function, potentially leading to a reduction in cardiovascular events in Asia.

## Conclusion

Serum UA levels are significantly associated with VSMC dysfunction as assessed by NID of the brachial artery in women. UA may be involved in functional and structural changes in VSMCs in women.

## Supplementary information

Online Supplement
