# Supplementary material for: Serum uric acid is independently associated with impaired nitroglycerine-induced vasodilation of the brachial artery in women
Source: Hypertens Res. 2024 Nov 14;48(1):6–14. doi: 10.1038/s41440-024-01972-5 (PMC11700840; doi:10.1038/s41440-024-01972-5)
Supplement: Supplementary file 1 — Online Supplement [file 41440_2024_1972_MOESM1_ESM.docx]

ONLINE SUPPLEMENT

**Serum Uric Acid Is Independently Associated with Impaired Nitroglycerine-induced Vasodilation of the Brachial Artery in Women**

Brief title: Uric acid and NID in women

Tatsuya Maruhashi, MD, PhD;^1^ Masato Kajikawa, MD, PhD;^2^ Shinji Kishimoto, MD, PhD;^1^ Takayuki Yamaji, MD, PhD;^1^ Takahiro Harada, MD, PhD;^1^ Aya Mizobuchi, MS;^1^ Shunsuke Tanigawa, MS;^1^ Farina Mohamad Yusoff, MD, PhD;^1^ Yukiko Nakano, MD, PhD;^3^ Kazuaki Chayama, MD, PhD;^4^ Ayumu Nakashima, MD, PhD;^5^ Chikara Goto, PhD;^6^ Yukihito Higashi, MD, PhD^1, 2^

^1^Department of Regenerative Medicine, Division of Radiation Medical Science, Research Institute for Radiation Biology and Medicine, Hiroshima University, 1-2-3 Kasumi, Minami-ku, Hiroshima, 734-8553, Japan

^2^Division of Regeneration and Medicine, Medical Center for Translational and Clinical Research, Hiroshima University Hospital, 1-2-3 Kasumi, Minami-ku, Hiroshima, 734-8551, Japan

^3^Department of Cardiovascular Medicine, Graduate School of Biomedical and Health Sciences, Hiroshima University, 1-2-3 Kasumi, Minami-ku, Hiroshima, 734-8551, Japan

^4^Department of Medicine and Molecular Science, Hiroshima University Graduate School of Biomedical Sciences, Hiroshima University, 1-2-3 Kasumi, Minami-ku, Hiroshima, 734-8551, Japan

^5^Department of Stem Cell Biology and Medicine, Graduate School of Biomedical and Sciences, Hiroshima University, 1-2-3 Kasumi, Minami-ku, Hiroshima, 734-8551, Japan

^6^Department of Rehabilitation, Faculty of general Rehabilitation, Hiroshima International University, 555-36, Kurosegakuendai, Higashihiroshima, 739-2695, Japan

**Correspondence**: Yukihito Higashi, MD, PhD, FAHA

Department of Regenerative Medicine,

Research Institute for Radiation Biology and Medicine, Hiroshima University

1-2-3 Kasumi, Minami-ku, Hiroshima, 734-8553, Japan

Phone: +81-82-257-5831 Fax: +81-82-257-5831

E-mail: [yhigashi@hiroshima-u.ac.jp](mailto:yhigashi@hiroshima-u.ac.jp)

**Supplementary Table 1.** Clinical Characteristics according to Serum Uric Acid Levels in Men

HDL indicates high-density lipoprotein; LDL, low-density lipoprotein; eGFR, estimated glomerular filtration rate; NA, not applicable.

*p* values for comparisons across the uric acid categories were performed with ANOVA for continuous variables and χ^2^ test for categorical variables.

|  | Uric acid categories, mg/dL | | | |  |
| --- | --- | --- | --- | --- | --- |
|  | ≤5.0 | 5.0 to ≤6.0 | 6.0 to ≤7.0 | 7.0< |  |
| Variables | (n = 192) | (n = 318) | (n = 284) | (n = 214) | *p* value |
| Uric acid, mg/dL | 4.26 ± 0.67 | 5.58 ± 0.29 | 6.50 ± 0.29 | 7.95 ± 0.85 | NA |
| Age, y | 62.7 ± 17.2 | 60.0 ± 17.0 | 56.6 ± 19.4 | 57.3±17.7 | 0.001 |
| Body mass index, kg/m^2^ | 22.8 ± 3.2 | 23.5 ± 3.2 | 24.7 ± 3.8 | 25.0 ± 4.0 | <0.001 |
| Systolic blood pressure, mm Hg | 127.7 ± 16.9 | 129.3 ± 18.0 | 128.2 ± 16.6 | 132.4 ± 20.7 | 0.03 |
| Diastolic blood pressure, mm Hg | 73.9 ± 10.9 | 77.3 ± 12.1 | 76.7 ± 11.2 | 81.0 ± 13.9 | <0.001 |
| Heart rate, bpm | 68.1 ± 12.5 | 68.4 ± 11.9 | 67.4 ± 10.8 | 69.2 ± 11.7 | 0.39 |
| Total cholesterol, mg/dL | 182.5 ± 33.8 | 183.7 ± 38.1 | 188.6 ± 34.5 | 191.5 ± 40.2 | 0.03 |
| Triglycerides, mg/dL | 113.4 ± 72.6 | 143.1 ± 94.8 | 164.9 ± 175.9 | 171.6 ± 134.2 | <0.001 |
| HDL-cholesterol, mg/dL | 59.7 ± 16.1 | 57.7 ± 16.0 | 55.2 ± 15.1 | 56.9 ± 16.8 | 0.02 |
| LDL-cholesterol, mg/dL | 103.6 ± 30.0 | 103.0 ± 33.7 | 108.2 ± 29.9 | 108.6 ± 35.8 | 0.10 |
| Glucose, mg/dL | 116.3 ± 47.5 | 113.9 ± 36.0 | 112.9 ± 37.5 | 112.5 ± 40.4 | 0.78 |
| eGFR, mL/min/1.73 m^2^ | 75.0 ± 22.4 | 72.4 ± 20.5 | 73.7 ±18.9 | 67.6 ± 21.3 | 0.001 |
| Hypertension, n (%) | 142 (74.0) | 245 (77.0) | 198 (70.0) | 183 (85.5) | <0.001 |
| Dyslipidemia, n (%) | 114 (59.4) | 221 (69.5) | 185 (65.1) | 165 (77.1) | 0.001 |
| Diabetes mellitus, n (%) | 64 (33.3) | 86 (27.0) | 80 (28.2) | 47 (22.0) | 0.08 |
| Coronary artery disease, n (%) | 40 (20.8) | 53 (17.4) | 56 (20.2) | 51 (23.8) | 0.35 |
| Cerebrovascular disease, n (%) | 22 (11.5) | 20 (6.3) | 25 (9.1) | 19 (9.0) | 0.24 |
| Smoking, n (%) | 137 (71.7) | 235 (74.4) | 214 (75.6) | 157 (73.4) | 0.81 |
| Chronic kidney disease, n (%) | 38 (19.8) | 67 (21.1) | 56 (19.7) | 77 (36.0) | <0.001 |
| Diuretics, n (%) | 11 (5.8) | 37 (11.7) | 45 (16.3) | 46 (21.8) | <0.001 |

**Supplementary Table 2**. Multivariate Analysis of the Relation Between Vascular Smooth Muscle Dysfunction and Variables in Men

|  | Model 1 | |  | Model 2 | |  | Model 3 | |  | Model 4 | |  | Model 5 | | Model 6 | |
| --- | --- | --- | --- | --- | --- | --- | --- | --- | --- | --- | --- | --- | --- | --- | --- | --- |
| Variables | OR (95%CI) | *p* value |  | OR (95%CI) | *p* value |  | OR (95%CI) | *p* value |  | OR (95%CI) | *p* value |  | OR (95%CI) | *p* value | OR (95%CI) | *p* value |
| Uric acid (mg/dL) | 1.05 (0.95-1.15) | 0.37 |  | 1.10 (0.996-1.22) | 0.06 |  | 1.04 (0.93-1.17) | 0.49 |  | 1.06 (0.95-1.18) | 0.32 |  | 1.04 (0.93-1.16) | 0.54 | 1.04 (0.93-1.17) | 0.47 |
| Age (years) | - | - |  | 1.04 (1.03-1.05) | <0.001 |  | 1.03 (1.01-1.04) | <0.001 |  | 1.02 (1.01-1.04) | <0.001 |  | 1.02 (1.01-1.04) | <0.001 | 1.02 (1.01-1.04) | <0.001 |
| Body mass index (kg/m^2^) | - | - |  | - | - |  | 1.08 (1.03-1.12) | <0.001 |  | - | - |  | 1.07 (1.03-1.12) | 0.002 | 1.07 (1.03-1.12) | 0.001 |
| Body mass index ≥25 (kg/m^2^) | - |  |  | - |  |  | - | - |  | 1.49 (1.09-2.03) | 0.01 |  | - | - | - | - |
| Hypertension (yes/no) | - | - |  | - | - |  | 1.81 (1.11-2.96) | 0.02 |  | 1.84 (1.13-2.99) | 0.01 |  | 1.76 (1.08-2.87) | 0.02 | 1.74 (1.07-2.84) | 0.03 |
| Dyslipidemia (yes/no) | - | - |  | - | - |  | 1.19 (0.84-1.69) | 0.33 |  | 1.27 (0.90-1.80) | 0.17 |  | 1.17 (0.83-1.66) | 0.37 | 1.19 (0.84-1.70) | 0.32 |
| Diabetes mellitus (yes/no) | - | - |  | - | - |  | 1.17 (0.85-1.62) | 0.34 |  | 1.21 (0.88-1.67) | 0.25 |  | 1.18 (0.86-1.63) | 0.31 | 1.17 (0.85-1.62) | 0.34 |
| Smoking (yes/no) | - | - |  | - | - |  | 0.62 (0.44-0.86) | 0.004 |  | 0.61 (0.44-0.86) | 0.004 |  | - | - | 0.62 (0.44-0.87) | 0.005 |
| eGFR (ml/min/1.73m^2^) | - | - |  | - | - |  | - | - |  | 0.99 (0.98-1.00) | 0.10 |  | 0.99 (0.98-1.001) | 0.09 | 0.99 (0.98-1.001) | 0.09 |
| Chronic kidney disease (yes/no) | - |  |  | - |  |  | 1.40 (0.98-2.00) | 0.07 |  | - | - |  | - | - | - | - |
| Coronary artery disease (yes/no) | - | - |  | - | - |  | 0.89 (0.61-1.30) | 0.54 |  | 0.87 (0.60-1.26) | 0.46 |  | 0.86 (0.59-1.25) | 0.42 | 0.91 (0.62-1.33) | 0.62 |
| Cerebrovascular disease (yes/no) | - | - |  | - | - |  | 2.55 (1.55-4.21) | <0.001 |  | 2.52 (1.54-4.15) | <0.001 |  | 2.72 (1.66-4.45) | <0.001 | 2.59 (1.56-4.22) | <0.001 |
| Diuretics (yes/no) | - | - |  | - | - |  | 0.96 (0.64-1.45) | 0.86 |  | 0.98 (0.65-1.47) | 0.92 |  | 0.98 (0.65-1.47) | 0.98 | 0.97 (0.64-1.46) | 0.88 |

OR, odds ratio; CI, confidence interval; eGFR, estimated glomerular filtration rate.

**Supplementary Figure 1**


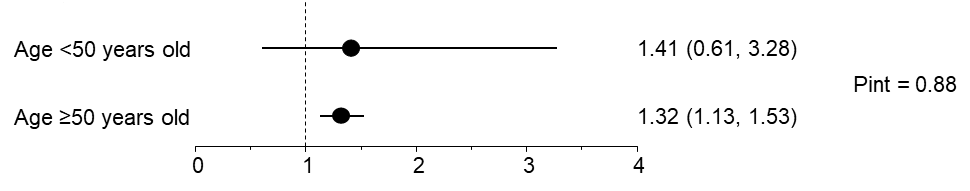


Odds ratios and 95% confidence intervals of 1 mg/dL increase in serum uric acid levels for vascular smooth muscle cell dysfunction defined as nitroglycerine-induced vasodilation <8.4% in subjects <50 years of age and subjects ≥50 years of age in women. Pint, *p*-value for interaction.

**Supplementary Figure 2**


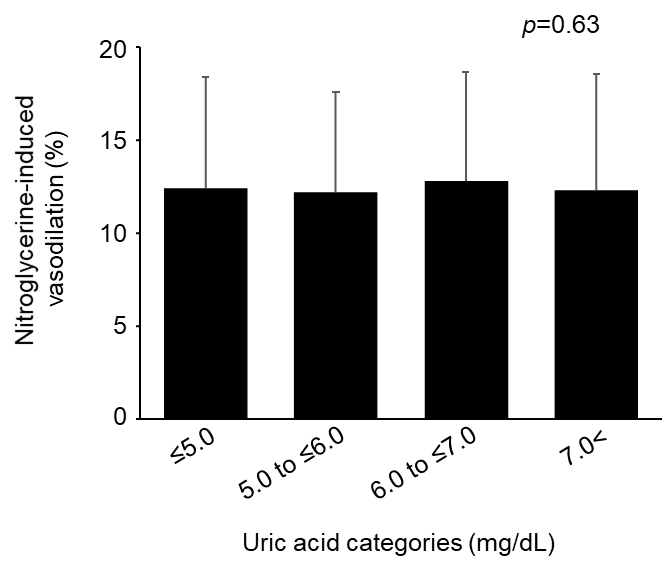


Bar graphs show nitroglycerine-induced vasodilation of the brachial artery categorized according to serum uric acid levels in men.

**Supplementary Figure 3**


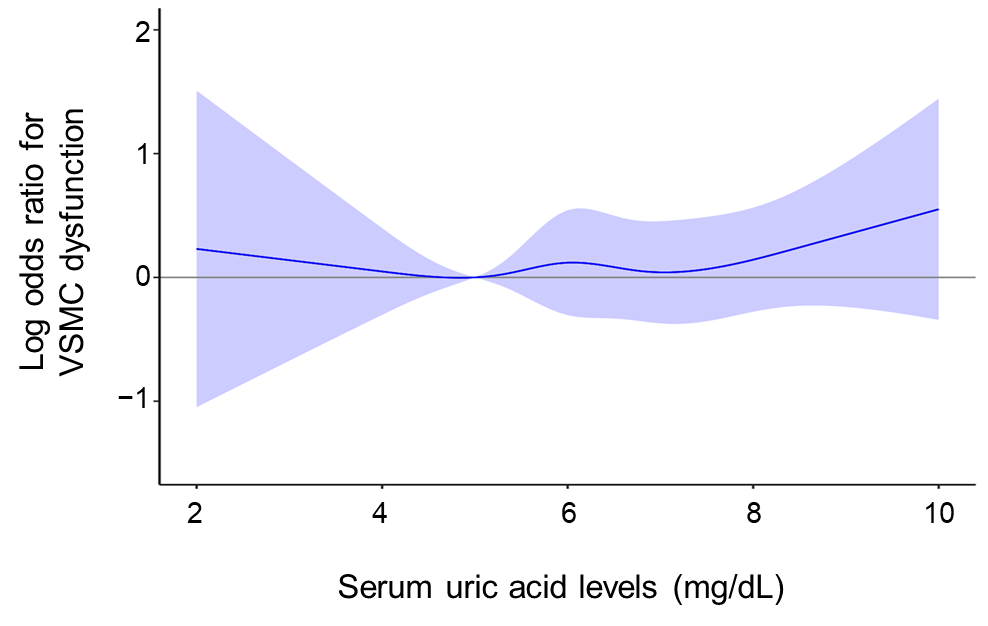


Adjusted cubic spline of the relationship between serum uric acid levels and vascular smooth muscle cell (VSMC) dysfunction in men. The adjusted model includes age, body mass index, hypertension, dyslipidemia, diabetes mellitus, smoking, estimated glomerular filtration rate, coronary artery disease, cerebrovascular disease, and diuretic use.
